# Deoxynivalenol Mycotoxin Inhibits Rabies Virus Replication In Vitro

**DOI:** 10.3390/ijms24097793

**Published:** 2023-04-25

**Authors:** Qian Liu, Qing He, Wuyang Zhu

**Affiliations:** NHC Key Laboratory of Biosafety, National Institute for Viral Disease Control and Prevention, Chinese Center for Disease Control and Prevention, Beijing 102206, China

**Keywords:** rabies, rabies virus, deoxynivalenol, apoptosis, mycotoxin

## Abstract

Rabies is a highly fatal disease, and it is vital to find effective ways to manage and control infection. There is a need for new effective antiviral drugs that are particularly effective treatments for rabies. Deoxynivalenol (DON) is known mainly for its toxicity, but at the molecular level, it can inhibit RNA and DNA replication, and there is increasing evidence that different doses of DON have a positive effect on inhibiting virus replication. Based on this, we evaluated the effect of DON on inhibiting the rabies virus in vitro. The inhibitory effect of DON on rabies virus activity was dose- and time-dependent, and 0.25 μg/mL of DON could inhibit 99% of rabies virus activity within 24 h. Furthermore, DON could inhibit the adsorption, entry, replication, and release of rabies virus but could not inactivate the virus. The inhibitory effect of DON on rabies virus may be achieved by promoting apoptosis. Our study provides a new perspective for the study of anti-rabies virus and expands the direction of action of mycotoxins.

## 1. Introduction

Rabies is a zoonotic viral disease caused by rabies virus (RABV), and 99% of cases are due to transmission through animal bites, particularly dogs [1]. The case fatality rate is nearly 100%, causing tens of thousands of deaths annually [2]. RABV is a neurotropic virus that mainly enters neurons through bitten skin and travels to the central nervous system to replicate [3]. RABV mainly encodes the five structural proteins: nucleoprotein (N), phosphoprotein (P), matrix protein (M), glycoprotein (G), and an RNA-directed RNA polymerase (L) [4]. As a single negative-stranded RNA virus, its genome transcriptional order is 3′-N-P-M-G-L-5′, and the transcriptional abundance of the five proteins decreases gradually [5]. The N protein is the most conserved and abundant structural protein, it can serve as an important component for the pathogenetic detection of rabies virus. The G protein interacts with host cellular receptors. After the complex of L and P protein is formed, the released RNP is used as a template for repeated transcription. The M protein affects the initiation of viral replication by regulating the threshold of viral N protein [6,7]. RABV, with its replicates at a low level in vitro and absence of cytopathic effect, has also been found to be highly pathogenic and currently has no effective treatment [8,9]. Therefore, the key to treating rabies is to eliminate or inhibit RABV in vivo, and the study of the inhibitory effect of RABV in vitro is a necessary prerequisite for exploring corresponding antiviral compounds [10]. Known antiviral compounds ribavirin [11,12] and T-705 [13] have shown significant anti-rabies virus effects in vitro. In addition, pyrimethamine [14], type-I interferon [15], and TMR-001 [16] have also been reported to inhibit the activity of RABV in vitro, but failed in the experiment of improving the survival rate of mice. Although it failed to show a therapeutic effect in a mouse model, ribavirin has also been used as a control for in vitro anti-rabies virus activity [17].

Deoxynivalenol (DON) is a type B trichothecene mycotoxin produced by Fusarium fungi [18]. DON is a common global pollutant of cereal grains and can pose a threat to human and animal health [19]. Although DON has a wide range of toxicities, there are significant differences in the biological activities at different doses; for example, different doses of DON can affect virus infection and replication [20]. Low concentrations (<560 ng/mL) of DON reduce porcine reproductive and respiratory syndrome virus (PRRSV) viral replication by inducing a proinflammatory cytokine environment and promoting early activation of apoptosis [21]. In addition, DON can inhibit DNA and RNA replication and protein synthesis, induce central neuroinflammation, and increase the permeability of the blood–brain barrier [22,23]. However, there is no in vitro model to determine the effect of DON on RABV infection. Therefore, the purpose of this study was to evaluate the inhibitory effect of DON on RABV in vitro.

Taken together, in this study, we investigated the antiviral activity of DON against RABV in vitro, and explored the mechanism of DON inhibiting virus replication.

## 2. Results

### 2.1. DON Exhibits Anti-Rabies Virus Activity in BHK-21 Cells

**Figure 1:**
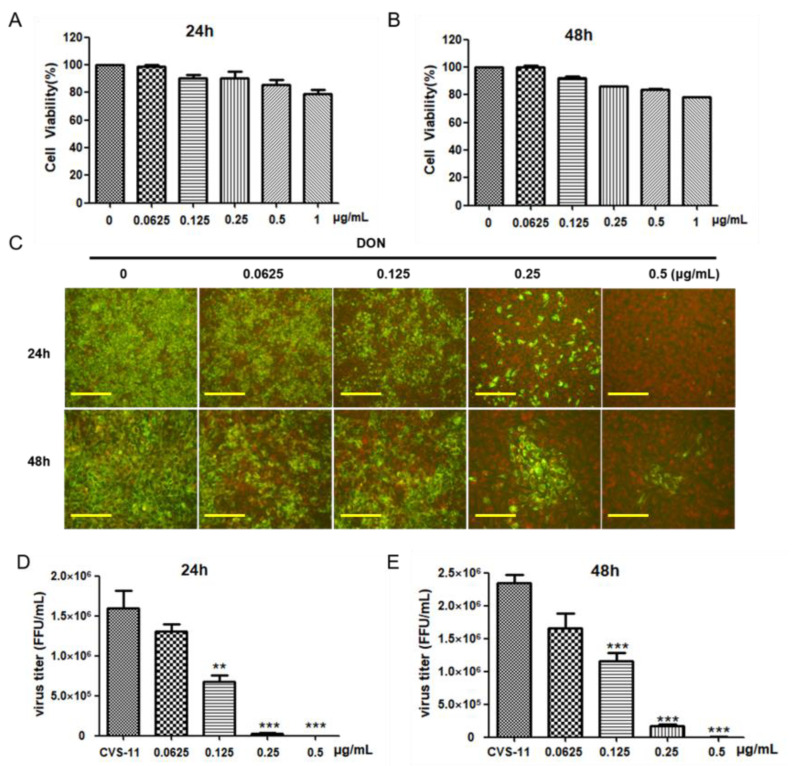
Effect of deoxynivalenol (DON) against rabies virus activity. BHK-21 cells were treated with different concentrations of DON for 24 (**A**) and 48 h (**B**), and cell viability was measured by a CCK-8 assay. Data are presented as % of the control (mean ± SD, n = 5). BHK-21 cells were infected with CVS-11 for 1 h in advance, and then treated with different concentrations of DON (0, 0.0625, 0.125, 0.25, and 0.5 μg/mL) for 24 and 48 h. The inhibitory effect of virus was observed via direct fluorescent antibody tests. The scales bar of 24 h and 48 h are 100 μm and 50 μm respectively (**C**). The virus titer of CVS-11 was measured using a fluorescent focus unit assay at 24 h (**D**) and 48 h (**E**). All results in the figure are representative of at least three experiments. Statistical significance was calculated via one-way ANOVA followed by Dunnett’s test, and significance was defined as *** *p* < 0.001, and ** *p* < 0.01.

To determine the potential anti-rabies virus activity of DON, we evaluated the cytotoxicity of DON via the CCK-8 assay. As shown in Figure 1A, the cell viability decreased with increasing concentration. Following treatment with 0.25 and 0.5 μg/mL of DON, the cell survival rate reached >80%. Prolonging the DON treatment to 48 h (Figure 1B), the cell survival rate at 0.5 μg/mL of DON reached 80%. On the basis of these results, we further conducted an antiviral assay to detect the inhibitory effect of DON on RABV. As shown in Figure 1C, with the increase in DON concentration, the green fluorescent focus of the virus gradually decreased, with negligible green fluorescent focus of the virus following treatment at 0.5 μg/mL of DON between 24 and 48 h. As shown in Figure 1D,E, treatment with 0.25 μg/mL of DON significantly reduced the virus titer while the inhibition rate reached 99% within 24 h. These results indicated that DON could inhibit RABV CVS-11 activity in a dose- and time-dependent manner.

### 2.2. DON Target Replication Phase of Viral Infection Cycle

**Figure 2:**
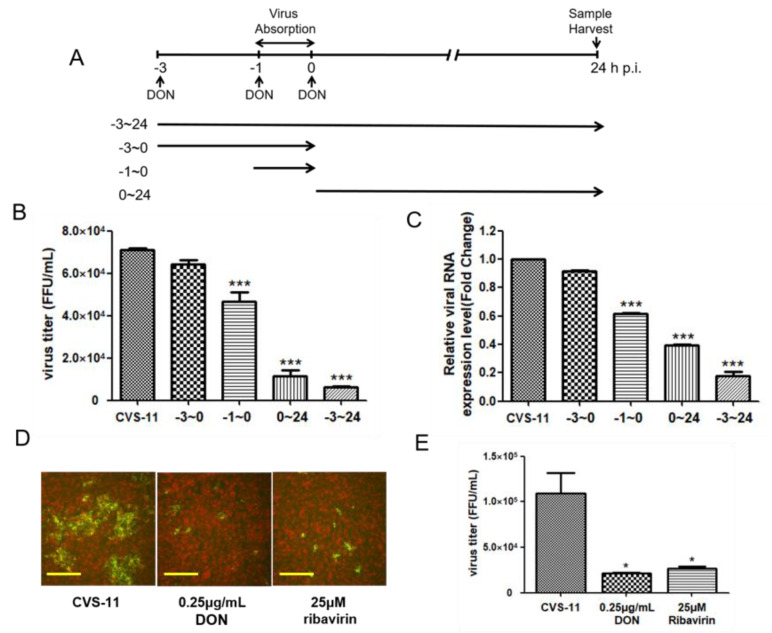
Treatment with DON inhibits CVS-11 at various stages of infection. (**A**) Schematic representation of the time-of-addition assay. (**B**) BHK-21 cells were infected with CVS-11 at a MOI of 0.01. Subsequently, DON (0.25 μg/mL) was added at the following time points: before virus entry (between −3–0 h p.i.) and during (−1–0 h p.i.) and following (0–24 h p.i.) virus adsorption. Infected cells were collectively harvested at 24 h p.i. Viral titers and RNA synthesis were analyzed using a fluorescence focus units (FFU) assay (**B**) and qPCR (**C**), respectively. Expression levels of viral RNA were initially normalized to those of β-actin mRNA. Moreover, the ratio measured in DON-treated cells was normalized to the RNA level of virus control (arbitrarily set to 1). CVS-11 infected BHK-21 cells were treated with 0.25 μg/mL of DON and 25 μM ribavirin for 24 h. CVS-11 viral infection and titers were measured using direct immunofluorescence assay, the scales bar are 100 μm (**D**) and fluorescence focus units assay(**E**). Data in bar charts are expressed as means ± standard error of the mean from at least two independent experiments. * *p* < 0.05, and *** *p* < 0.005.

We designed a schematic to identify the stage at which DON inhibits the viral replication cycle (Figure 2A) [24]. As shown in Figure 2B, compared with the simulated control group, the inhibitory effect of DON in the interval from −3 to 24 h p.i. was clearly apparent, and the inhibitory effect was >99%. During the virus replication stage in the interval from 0 to 24 h p.i., the inhibitory effect of DON on the virus was >90%, whereas in the early infection stage of the virus (in the interval from −1 to 0 h p.i.), the inhibitory effect of DON was approximately 30%. Simultaneously, we found that DON had a similar trend on the replication stage of CVS-11 at the viral RNA level. These results suggest that DON mainly inhibits the replication stage of the virus and has an inhibitory effect during the early stage of infection. In addition, using DFA and fluorescent focus transformation experiments, we found that 0.25 μg/mL of DON and 25 μM ribavirin had the same inhibitory effect on the replication stage of RABV. Together, these results indicated that DON primarily inhibits the replication phase of the CVS-11 infection cycle.

### 2.3. DON Can Inhibit the Binding, Entry, and Release of Rabies Viruses but Cannot Directly Inactivate Viruses

**Figure 3:**
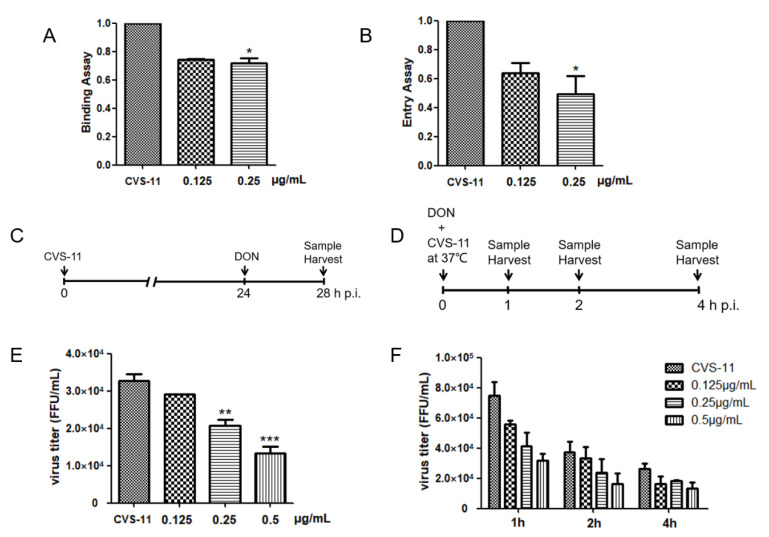
Adsorption, binding, release, and inactivation of CVS-11 virus treated with DON. (**A**) Binding assay, (**B**) entry assay, (**C**) experimental scheme for release assay, (**D**) experimental scheme for inactivation assay, (**E**) release assay, and (**F**) inactivation assay. Briefly, CVS-11 virions were directly treated with DON (0, 0.125, 0.25, and 0.5 μg/mL) at 37 °C. Part of the inoculum was removed at the indicated time points and serially diluted for the fluorescence focus units assay to determine the number of infectious rabies virions. One-way ANOVA was followed by Dunnett’s test. * p < 0.05, ** p < 0.01, *** *p* < 0.001, as compared with the CVS-11 control.

Based on the above results, we identified a suppressive effect of DON early in RABV infection. Therefore, we performed further experiments that included inhibition of RABV binding and entry. In the binding experiment, we pre-incubated CVS-11, DON, and BHK-21 cells at 4 °C for 1 h, then replaced the medium with normal culture medium for 24 h, followed by RNA extraction after cell lysis. The adsorption inhibition effect of 0.25 μg/mL of DON was approximately 25% (Figure 3A). In the entry experiment, we pre-incubated CVS-11 and BHK-21 cells at 4 °C for 1 h, incubated with DON at 37 °C for 1 h, and then replaced with normal medium for 24 h. As shown in Figure 3B, increasing the concentration of DON toxin increased the inhibition effect of CVS-11 virus entry, and this reached 50% at 0.25 μg/mL of DON. In the release experiment, the titer of CVS-11 showed a downward trend with the increase in concentration of DON, and 0.5 μg/mL of DON decreased the titer by >50%. Additionally, we performed virus inactivation experiments to determine whether DON could directly target virions. We set up different time treatments and different concentrations of DON to incubate directly with the virus. These results showed that effects of DON to inhibit infectious RABV virions in the inoculum were dose-dependent after 1–2 h treatment. Moreover, 0.5 μg/mL of DON inactivated RABV virions by approximately 50% after 1–2 h incubation. With a 4 h treatment, the virucidal effect of DON was not apparent, and the drug did not exhibit a dose effect, which may be related to the decrease in the titer of the virus control itself under treatment at 37 °C (Figure 3E,F). These results demonstrated that DON can inhibit the adsorption, entry, and release of viruses but cannot directly inactivate viruses.

### 2.4. DON Inhibits the Replication of SC-16 and CTN-1 Strains

**Figure 4:**
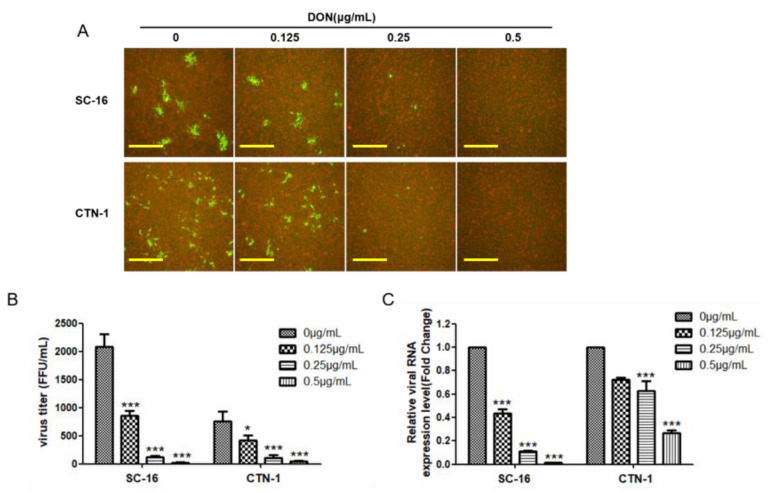
Effect of DON on SC-16 and CTN-1 replication. Different strains of rabies virus were treated with different concentrations of DON. (**A**) Direct immunofluorescence assay was used to detect virus infection, and the scales bar are 100 μm, (**B**) fluorescence focus units assay was used to detect virus titer, and (**C**) qPCR was used to detect the relative expression of the N gene in different strains. BHK-21 cells were infected with CTN-1 and SC-16 at a MOI of 0.01. One-way ANOVA was followed by Dunnett’s test, * *p* < 0.05, *** *p* < 0.001.

The above results proved that DON can inhibit the replication of RABV CVS-11. To determine the inhibitory effect of DON on other RABV strains, we chose street virus strain SC-16 and vaccine strain CTN-1 for experiments. After the BHK-21 cells were infected with the virus, they were treated with 0.125, 0.25, and 0.5 μg/mL of DON for 24 h. The SC-16 and CTN-1 strain fluorescence foci decreased gradually compared with those in the control group, and the virus fluorescence foci decreased to almost zero upon treatment with 0.5 μg/mL of DON (Figure 4A). As shown in Figure 4B,C, increasing the concentration of DON gradually decreased the virus titer of SC-16 and CTN-1 strains and the expression of the virus N gene. In addition, the inhibitory effect of DON on the SC-16 strain was more significant than that of vaccine strain CTN-1, and treatment with 0.125 μg/mL of DON had a 60% inhibitory effect on SC-16 whereas this was only 30% on CTN-1. This difference may be related to the virulence of the virus (Figure 4B). Together, these results indicated that DON has anti-rabies virus activity against street and vaccine strains of rabies.

### 2.5. DON May Affect the Replication of Rabies Virus through Apoptosis

**Figure 5:**
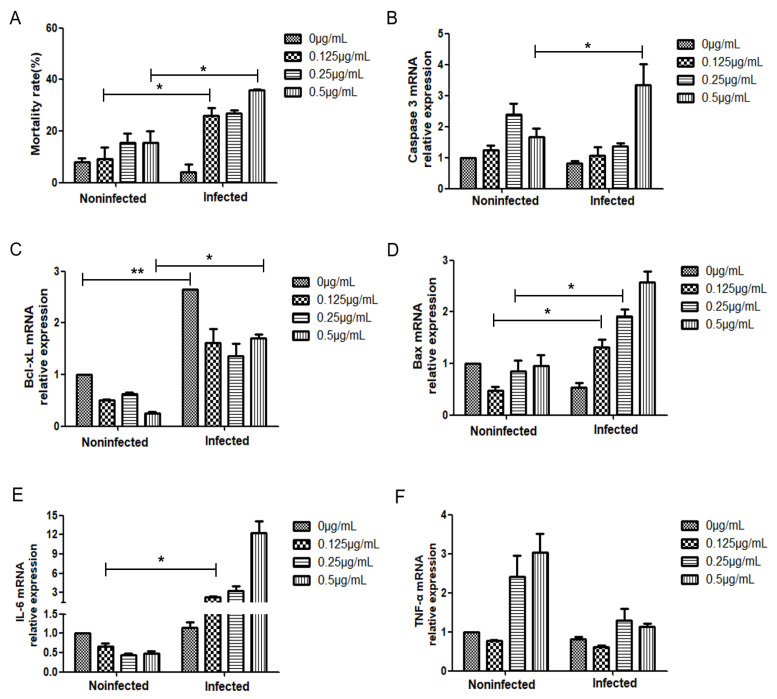
The related indicators of cell apoptosis after DON toxin infection with CVS-11 strain were detected. Noninfected or infected BHK-21 cells were treated with increasing concentrations of DON (0.125, 0.25, or 0.5 μg/mL). Cell mortality was evaluated by LDH release for 24 h following DON treatment (**A**). The relative expression levels of Caspase 3 (**B**), Bcl-xL (**C**), BAX (**D**), IL-6 (**E**), and TNF-α (**F**) mRNA were determined using qPCR in uninfected and infected cells. Data labeled with superscripted “*” indicate a significant difference between data sets (* p < 0.05, ** *p <* 0.01). Results are representative of three independent experiments for each type.

Studies have reported that DON toxin can induce the release of LDH [23]. To explore the specific role of DON in inhibiting RABV replication, we first evaluated the cell death rate by detecting the changes in LDH release in BHK-21 cells before and after infection with RABV. First, the cell death rate in the virus-infected control group was reduced compared with that in the uninfected cell control, which also supports the previously mentioned replication strategy of rabies. In addition, in both the infected and the uninfected groups, an increase in the concentration of DON produced an increase in the release of LDH and an increase in the cell death rate. Treatment with 0.25 μg/mL of DON caused cell death rates of approximately 15% and 25% in the uninfected and virus-infected groups, respectively. Subsequently, we assessed the mRNA levels of apoptosis-related proteins caspase 3 (Figure 5B), BAX, and Bcl-xL before and after virus infection and found that the level of caspase 3 mRNA was increased in the group treated with DON compared with that in the control group. The levels of BAX mRNA in the infection group gradually increased compared with those of the control group while those of Bcl-xL mRNA trended downward (Figure 5C,D). In addition, we assessed the mRNA expression of proinflammatory factors IL-6 and TNF-α (Figure 5E,F), and the results showed that, compared with that in the control group, the expression of IL-6 in the infected group increased with the increase in DON concentration, while that in the uninfected group declined. The expression of TNF-α mRNA increased with the increase in DON concentration in the uninfected group and decreased in the infected group compared with that in the control group. Together, the above results indicated that DON may affect the replication of RABV through apoptosis.

## 3. Discussion

Rabies has a significant morbidity and mortality rate, and RABV replication can cause severe damage to the host cell metabolism as well as prevent apoptosis and evade innate and adaptive immune responses. For these reasons, although DON is widely recognized as a toxin, the associated mechanism of action also includes inhibiting RNA, DNA, and protein synthesis, and can selectively drive a group of immune and inflammatory responses and intracellular signal transduction systems, such as apoptosis, as well as other signaling pathways [25]. Therefore, as the pathogenic mechanism of RABV is countered by the molecular mechanism of DON, we chose to evaluate the anti-rabies virus activity of DON. Herein, we found that DON has anti-rabies virus activity in vitro and can inhibit the adsorption, entry, and release of RABV at a specific concentration. In addition, the inhibitory effect of DON on RABV may be caused by the promotion of apoptosis [26]. These findings not only broaden the spectrum of anti-rabies virus treatments but also provide new insights into the application of DON.

In earlier studies, low concentrations of DON were found to inhibit HSV-1 replication, and this inhibition occurred after virus adsorption rather than before or during virus adsorption to host cells [27]. This is similar to our findings that DON mainly inhibits the replication of RABV and has an inhibitory effect on the entry phase of the virus. In addition, PRRSV replication was found to be significantly inhibited when treated with 140–280 ng/mL of DON. However, when studying the effect of DON on porcine epidemic diarrhea virus (PEDV), low concentrations of DON increased PEDV infection and replication, which was consistent with the increased replication of CVS-11 virus here when treated with 0.03125 μg/mL of DON (results not shown) [28].

In the time-of-addition assays, we found that DON had no significant effect on viral infection after pre-incubating cells (from −3 to 0 h p.i.), but in the early stage of viral infection (from −1 to 0 h p.i.), the inhibitory effect reached 30%. Further experiments revealed that DON had an inhibition effect both at the adsorption and entry stages of RABV. It needs to be further investigated whether DON inhibits the entry of RABV into cells by altering the viral receptor on BHK-21 cells. Subsequently, we used ribavirin as a positive control and found that 0.25 μg/mL of DON had a similar effect to 25 μM ribavirin [11]. In addition, using virus inactivation experiments, we found that the decrease in activity of RABV over time may be related to the sensitivity of RABV to temperature [29]. In the virus replication cycle of release experiment, the release of RABV decreased with the increase of the concentration of DON, which may be related to the M protein of RABV [30], and further exploration is needed to follow up this result.

In addition to studying the effect of DON on the replication of the CVS-11 strain, we also studied the effect of DON on the replication of the street virus strain SC-16 and the vaccine strain CTN-1. Compared with the effect on the replication of CTN strains, the inhibitory effect of a low concentration of DON on street virus strains was more apparent. This result may imply that DON has a stronger inhibitory effect on virulence and that this inhibitory effect may be induced by apoptosis [31]. RABV infection maintains RABV gene expression below threshold levels by interfering with proapoptotic factors, causing neuronal dysfunction rather than neuronal death. However, virulent strains of rabies virus ensure their spread by preventing apoptosis, whereas attenuated strains can cause cell apoptosis [32]. Consequently, low concentrations of DON have no significant inhibitory effect on the CTN-1 strain, which is similar to the experimental results in this study. In addition, virus titers of Figure 4B,C in control samples without DON treatment varied greatly, so it may also be related to the adaptation of the virus strains to cells, and further experiments are required to verify this.

Savard et al. found that low concentrations of DON inhibited PRRSV replication and reduced viral replication by inducing a proinflammatory cytokine environment and promoting the early activation of apoptosis. Apoptosis is an important host defense mechanism as it interrupts viral replication and eliminates virus-infected cells [33]. Additionally, DON induces apoptosis by disrupting cell membranes to promote the release of LDH [34,35]. Therefore, we also examined the effect of DON treatment on the level of LDH released as well as on the expression of apoptosis and immune-related genes before and after CVS-11 infection, and showed that DON treatment of infected BHK-21 cells increased LDH release and induced apoptosis [34]. The effect on the release of LDH was similar to that on the level of mRNA of apoptosis-related genes for caspase 3, BAX, and Bcl-xL [36]. Proinflammatory factors can induce apoptosis to protect cells [37], and the changes in the expression of IL-6 and TNF-α mRNA showed that DON may be involved in the induction of several inflammatory markers. DON has dual effects of immunosuppression and immune stimulation. Simultaneously, DON can open the blood–brain barrier by activating the expression of immune factors, such as inflammatory factor IL-1β, and this effect should be further explored using in vivo models [38].

Although rabies can be prevented, it cannot be treated. This study evaluated the antiviral effect of DON and further explored the antiviral mechanism, which is valuable in understanding the pathogenic target of rabies and enabling improved blocking of the spread of RABV. In conclusion, we demonstrate that DON can inhibit RABV activity and that this inhibition may be mediated through apoptosis. Our results support the hypothesis that the apoptotic pathway may be the main pathway whereby DON blocks RABV transmission.

## 4. Materials and Methods

### 4.1. Cell Culture, Virus, and Compounds

Baby hamster kidney (BHK)-21 cells (American Type Culture Collection, Certified Cell Line-10) were cultured at 37 °C in Dulbecco’s modified Eagle’s medium (DMEM, Invitrogen, Carlsbad, CA, USA) containing 10% fetal bovine serum (PAN-Biotech, Aidenbach, Germany), 100 U/mL of penicillin G, and 100 g/mL of streptomycin (GIBCO, 15140-122, USA). BHK-21, as a routine cell infected by RABV, can be used to study the interaction between RABV and cells [39,40]. Dimethyl sulfoxide, DON, and ribavirin were purchased from Sigma–Aldrich (St. Louis, MO, USA).

The challenge virus standard 11 (CVS-11), SC-16, and CTN-1 strains (preserved in the Rabies Department, National Institute for Virus Disease Control and Prevention, Chinese Center for Disease Control and Prevention) were propagated in BHK-21 cells and stored at −80 °C. Virus titers were determined as the 50% tissue culture infective doses (TCID_50_) per mL by direct immunofluorescence assay (DFA).

### 4.2. Quantitative Reverse Transcription PCR (qRT-PCR)

RNA was extracted with TRIzol reagent (Invitrogen, Carlsbad, CA, USA). cDNA was reverse transcribed from 1 μg of RNA with M-MLV Reverse Transcriptase (Promega, Madison, WI, USA), oligo dT (6mer), and 10 mM dNTPs (TaKaRa, Dalian, China). Real-time PCR was performed on a BioRad CFX96 real-time PCR detection system using GoTaq qPCR Master Mix (Promega, Madison, WI, USA). Fold variations between RNA samples were calculated using the 2^−ΔΔCT^ threshold cycle method after normalization to the amount of β-actin mRNA. The procedure for fluorescence quantification was 95 °C for 3 min, followed by a program cycle of 40 repetitions of 95 °C for 10 s, 58 °C for 20 s, and 72 °C for 20 s. The primer sequences of each gene are shown in Table 1.

### 4.3. Direct Fluorescent Antibody Test

For DFA staining [41], cell supernatants were discarded, and cells were washed with phosphate-buffered saline (PBS) three times and then fixed in acetone at −20 °C for 10 min, followed by staining with mouse FITC-labeled RABV antibody (Fujirebio, Tokyo, Japan) for 30 min or 1 h. Cells were observed using fluorescence microscopy (OLYMPUS, 1X51, Japan).

### 4.4. Fluorescent Focus Unit (FFU) Assay

The virus supernatant was diluted five-fold for 12 gradients with serum-free DMEM in a 96-well plate. BHK-21 cells were mixed with viruses in 37 °C for 48 h. The virus titer was detected via DFA. The number of fluorescent foci < 20 in each well were recorded. The virus titer was calculated using the following formula [42],

Virus titer (fluorescent focus unit [FFU]/mL) = (mean number of fluorescent foci in the last 4 wells × dilution factor × 1000)/50.

### 4.5. Antiviral Assay

BHK-21 cells were seeded into 24-well plates at a density of 2 × 10^5^ cells per well at 37 °C in 5% CO_2_. When the cell concentration reached approximately 70–80% confluence, they were infected with CVS-11 (multiplicity of infection [MOI] = 0.1) at 37 °C for 1 h. After washing with PBS, fresh culture medium was added to the cells containing various concentrations of DON (0.0625–0.5 µg/mL) or ribavirin (25 μM) and incubated for 24 or 48 h. The supernatant was collected for virus titer determination, and the antiviral effects were determined via DFA and FFU assay.

### 4.6. Time-of-Drug-Addition Assay

To determine the effects of DON on different stages of CVS-11 virus infection in BHK-21 cells, the CVS-11 virus was absorbed to BHK-21 cells between −1 and 0 h at an MOI of 0.01. DON (0.125, 0.25, and 0.5 μg/mL) was administered at four time points: between −3 and 0 h (preinfection), between −1 and 0 h (coinfection), between 0 and 24 h (postinfection), and between −3 and 24 h (full infection). Cell lysates from each treatment were harvested at 24 h for qRT-PCR. The supernatant was collected for virus titer determination.

### 4.7. Viral Binding, Entry, and Release Assay

For the binding assay, BHK-21 cells were cooled for 1 h at 4 °C and then infected with CVS-11 in the presence of different concentrations of DON for 1 h at 4 °C. After washing with PBS, cells were cultured with fresh medium for 24 h at 37 °C. After that the cells were lysed, and RNA was extracted for further analysis using qRT-PCR. For the entry assay, cells were incubated with CVS-11 for 1 h at 4 °C and switched to 37 °C for 1 h in the presence of different concentrations of DON after washing three times. Then cells were washed with PBS and incubated for another 24 h at 37 °C, and RNA was extracted for further analysis using qRT-PCR. For the release assay, cells were incubated with CVS-11 for 24 h at 37 °C. After washing with PBS, cells were cultured with different concentrations of DON for another 4 h at 37 °C. After different treatments, the supernatant was collected and titrated using TCID_50_.

### 4.8. Viral Particle Inactivation Assay

CVS-11 was diluted to 10^5^ FFU/mL with serum-free medium, and various concentrations of DON toxin were added at the indicated final concentrations. The mixtures were incubated at 37 °C for 1–4 h. At each time point, 500 µL of the mixtures was removed for virus yield determination. Three-fold serial dilutions of the mixtures were prepared, and the number of infectious CVS-11 in the inoculum was determined using the FFU assay on BHK-21 cell monolayers.

### 4.9. Cell Viability Assay

BHK-21 cells were seeded into 96-well plates at a density of 10^5^ cells per well until the confluence reached 80%, followed by treatment with DON at concentrations of 0.0625, 0.125, 0.25, 0.5, and 1 μg/mL for 24 h and 48 h, respectively. At each time point, cell viability was determined using the CCK-8 assay (Beyotime, Shanghai, China) in accordance with the manufacturer’s instructions. In brief, cells were treated with 10 μL of CCK-8 reagent per well for 1 h at 37 °C, and the absorbance was measured at 450 nm. All tests were performed three times.

### 4.10. Determination of Cell Mortality

BHK-21 cells were seeded into 96-well plates followed by DON treatment. Intracellular lactate dehydrogenase (LDH) was detected using LDH detection reagents (Promega, Madison, WI, USA). The LDH levels were measured using a fluorescent microplate reader (Paradigm Molecular Devices, SpectraMax Paradigm, Sunnyvale, CA, USA) at an absorbance of 450 nm.

### 4.11. Data Statistics and Analysis

Statistical software SPSS 20.0 (IBM, New York, NY, USA) was used to analyze the data. Data analysis was performed using GraphPad Prism 5 (GraphPad Software, San Diego, CA, USA). All data are expressed as the mean ± standard deviation (SD). For multiple groups, statistical significance was evaluated by one-way analysis of variance (ANOVA). When *p* < 0.05, the difference was significant. *: *p* < 0.05; **: *p* < 0.01; ***: *p* < 0.001.

## Figures and Tables

**Table 1:** Sequences of the primers used for qRT-PCR.

Gene (Abbreviation)	Sequence (5′→3′)
*β-actin*	F: GTGCTATGTTGCTCTAGACTTCG
	R: ATGCCACAGGATTCCATA
*Rabies virus N gene*	F: AATCTCACCGCAAGGGAAGC
	R: ATGCAGCAATAACCGTCGCA
*Caspase 3*	F: GGAATGGCATGTCGATCTGGT
	R: ACTGTCCGTCTCAATCCCAC
*BAX*	F: CCCGAGAGGTCTTTTTCCGAG
	R: CCAGCCCATGATGGTTCTGAT
*Bcl-xL*	F: TATTGGTGAGTCGGATCGCA
	R: CTCTCAGCTGCTGCATTGTT
*IL-6*	F: ACTCCCTCTCCACAAGCGCCTT
	R: TGGCATCTTCTTCCAGGCGTCCC
*TNF-α*	F: GCCCACGTTGTAGCCAATGTCAAA
	R: GTTGTCTTTCAGCTTCACGCCGTT

## Data Availability

The data presented in this study are available on request from the corresponding author. The data are not publicly available due to privacy or ethical restrictions.
